# Dutrochet's Discoveries in Vegetable and Animal Physiology

**Published:** 1829-07-01

**Authors:** 


1. L'AGENT IMMÉDIATE DU MOVEMENT VITAL, DEVOILÉ DANS SA NATURE, ET DANS SON MODE D'ACTION CHEZ LES VÉGÉTAUX ET LES ANIMAUX. Par *M. Dutrochet*. Paris, 1826.

2. NOUVELLES RECHERCHES SUR L'ENDOSMOSE ET L'EXOSMOSE, SUIVIES DE L'APPLICATION EXPERIMENTALE DE CES ACTIONS PHYSIQUES A LA SOLUTION DU PROBLEME DE L'IRRITABILITÉ VÉGÉTALE, &c. Par *M. Dutrochet*. Paris, 1828.

THE hackneyed apophthegm, *in extremis ad extrema*, has a very extensive application; for, whether we speak with allusion to the ten thousand fancies of female dress and the ten thousand forms of female fancy, or to the yearly revolutions which occur in politics and the daily recantations which obtain in science, our *dictum sapientiæ* is equally appropriate. Were it possible to confine inconsistency to the wardrobe or the toilet, to the promenade or the drawing room, the fop might be tolerated to suit his coat to his own taste, and to wear it either off or on, as fashion dictated; but when we see sciences, which should be constructed upon certain rules and studied under certain principles, subjected to the caprice of every taste, and tortured into every accommodating form to chime in with the eccentricities of monomaniasm, silence is generally impossible, and yet remonstrance is frequently of little use. Pathology is the theme of one age, physiology is the hobby of another; one book makes way for hundreds, and as soon as a President or Professor writes upon a certain point, popularity is forthwith stamped upon the subject, interest flows in another channel, the cue must be grasped by the other end, the child must be propitiated with another rattle, and a host of satellites may shortly be expected.

These *literary trade-winds*, it cannot be denied, may be of some use, but it is questionable, whether the plethora, which they induce in one market should be considered a sufficient counterbalance for the poverty they occasion in another, or whether the demand, which is thus made upon a popular commodity, may not hold out encouragement for adulteration, as well as for excess.

While it has been both our principle and practice to make every subject introduced into our pages bear as directly as possible upon therapeutical medicine, the *ancillary* sciences have not been, and ought not to be, overlooked; and in this our pathological day, it will be refreshing, both for our readers and ourselves, to leave, for a time, the lazar house and the dissecting table, and enter with the physiologist into a department of our profession, which seldom fails to make ample compensation for any lack of practical tendency it may be taxed with, in the delight and improvement it affords. Dutrochet has been for some time known to the literary world as a zealous and successful naturalist. His anatomical and physiological researches into both the animal and vegetable kingdoms have cast very considerable light upon much of their minute structure and many of their obscure functions. But it is not our design, at present, to review his botanical labours *en masse*; such a task, to be properly performed, would require us to embark into the subject of vegetable anatomy to an extent equally incompatible with our space and time. Our present article, shall, therefore, be confined to the investigation of two points of great interest, which the author proposes in the form of queries:—*What is the course of the sap in vegetables, and what are the powers by which it circulates?* And, by following him through his investigations on these questions, we shall have an opportunity of extracting much interesting matter, convinced, as we are, that the novelty of many of the principles which are developed is only equalled by the importance of the conclusions to which they lead, and by the variety of subjects to whose illustration they may be applied.

In the first of the volumes cited at the head of this review, Dutrochet opens his investigation of the first question—*what is the course of the sap in vegetables?* by observing, that it is generally admitted that the sap both ascends from the roots and descends from the leaves, and that, while its upward movement is too obvious to admit of doubt, the evidence by which its descent is ascertained consists of facts which can neither be clearly explained nor understood, until the route which it pursues in its journey to the leaves has been previously examined. By cutting a vine-branch across in the beginning of Spring, a watery fluid will be seen to exude from the extremities of small vessels, which are called by Decandolle lymphatic tubes, by Mirbel false tracheæ, and, by our author corpusculiferous tubes. These vessels run in straight lines, never anastomose, and have no valves. In young plants they are interiorly divided by transverse laminæ into elongated cells, which have no direct communication with each other; but when vegetation advances, their organization changes, these partitions are removed, leaving nothing as a vestige of their existence but small circular swellings on the interior of the vessel, and what was once a concatenation of cells becomes a continuous tube. In 1733, it was asserted by Sarrabat, that no sap ascended in either the bark or pith; Bonnet entertained the same idea, and experiments, variously modified, have convinced Dutrochet that Sarrabat and Bonnet were correct. But while lymphatic tubes are confined to the wood, and lymphatic sap is supposed to ascend in this portion of the plant exclusively, some have considered the alburnum the principal medium of its transmission, while others, as Coulon, have ascribed this office to the duramen. There are many facts, however, to shew that the sap ascends both by the duramen and alburnum, and the origin of a contrary sentiment may, probably, be traced to the difficulty of explaining, in accordance with our ordinary theories of vegetable circulation, the ascent of a fluid in vessels completely *lignified,* whose walls can scarcely be supposed to possess either contractility or irritability. Dutrochet attaches to these lymphatic tubes the function of circulating all the lymphatic or ascending sap, and, in opposition to much authority, maintains that the tracheæ perform a very different office. These vessels are met with only in the medullary sheath of dicotyledinous plants, so that, in monocatyledinous vegetables, the lymphatic tubes must be the unaided means whereby the ascent of the sap can be effected; and, when we consider that the tracheal vessels have not been discovered in the route of even dicotyledinous plants, it is natural to infer that their functions and those of the lymphatic tubes, which are as copious in the roots as in the stem, can have little in common. It is true that, on immersing the inferior end of a cut twig into a coloured liquid, both tracheal and lymphatic vessels will be found to take up the fluid; but there is this material difference between their actions, while, in the latter, the fluid will rise to the top of the plant, or, in other words, to the extremity of the vessels in which it is contained, it will ascend no higher than its own surface in the former. This experiment is tolerably decisive of the point, that some other function than that of raising the sap pertains to the tracheæ; what this function is has been much agitated, and, if at all illustrated, must be admitted to remain as yet obscure. Some naturalists will have it, that the ancient doctrine has been undeservedly rejected, and that there are many reasons for believing they contain air; others say they assist in circulating the sap; and some maintain that they are very analogous to the tracheæ of insects, and that they are the media by which an *influence vivifiante* received from the leaves is carried through the interior of the plant.

The leaves, the flowers, and the fruit are the parts to which the ascending sap ultimately tends. In these organs much of this fluid is evaporated and given to the atmosphere, while some of it is converted into a nutritious liquid, suited for the support of vegetable life. That it is mainly in the leaves that this conversion takes place is evident from many facts, and from none more clearly than this, that whenever a tree is deprived of its foliage growth ceases, vegetation gradually declines, and its fruit soon falls unripened to the ground. As soon, then, as this assimilating process has been perfected, and the crude lymph, which was extracted by the roots from the soil, has acquired those properties which adapt it for the discharge of its specific functions, the circulation assumes an opposite direction, and the elaborated sap commences its descent. The truth of this statement is easily established. By cutting out a circular piece of bark from the branch of a tree, it will be found that the superior cut surface alone, which is nearest to the leaves, will grow. This circumstance has given origin to the doctrine, that the elaborated sap descends by the bark only; others, as Knight, imagine that it can circulate through the alburnum also, when any impediment, by which the life of the plant becomes endangered, is given to its progress along the bark; and Dutrochet concludes that, while the ligneous tissue forms the easiest channel for its passage, it may be transmitted by the bark, but that it does not owe its circulation to an inverted action of the lymphatic tubes, as Knight supposes, but to the *clostres* which abound in both the bark and wood.

"The elaborated sap," he observes, "in reality descends in herbaceous plants both by their cortical and central systems, and this is one reason why we may expect it to follow the same course in ligneous vegetables. In these its descent is principally conducted in the young and, as it were, *herbaceous* tissue of the new bark and new alburnum, but it is probable that the entire alburnum, as well as some of the outer layers of the duramen, contribute in some degree to its transmission. The successive transformation of laminæ of alburnum into duramen seems to prove, that the oldest alburnum, or youngest duramen, still receives the elaborated sap, since this conversion of alburnum into duramen is the result of a chemical action which depends upon nutrition, an action so slow in its progress as to require several years for its completion. In my *Recherch. sur la Struct. intim. des Anim. et des Veget.* I have shown that this change consists in the hardening and coloration of a substance contained in the closters, which is formed from the elaborated sap, or is in fact the sap itself, rendered concrete by evaporation. I am, therefore, induced to regard these closters, which abound in the wood and bark, as the organs by which the elaborated sap is transmitted. This transmission is effected, as we shall hereafter see, by a particular action of the closters, which have no direct communication among themselves, but exert a suction power on each other, which draws this fluid through their walls. Finally, I have ascertained that the course of the elaborated sap is not always downward, since it ascends into the peduncles of the fruit, is the cause of their development, and likewise forms the shoots and buds."

It is well known, especially by bark-peelers, that in dicotyledinous plants, during the months of Spring and August, a quantity of fluid is effused between the wood and bark; but it has been hitherto uncertain whether it issues from the lymphatic tubes, is elaborated sap, or circulates through a channel and in a direction of its own. Dutrochet's reasoning on this difficulty is highly ingenious, and probably correct. He observes that, since this effusion occurs while the lymphatic sap is ascending in the greatest quantity, it might be natural to look for its origin to the lymphatic tubes; but, independent of the fact that this effusion has been witnessed during Winter, while no lymphatic sap was in motion, there are several circumstances unfavourable to this view. The sap thus effused seems to exude from the ligneous substance of the plant in an horizontal direction, and no horizontal machinery is known, through which this passage could be effected, but the *medullary radii* which exclusively belong to dicotyledinous plants, and traverse the ligneous tissue from the medullary sheath to the exterior surface of the alburnum, over which this fluid is effused. But it is in dicotyledinous plants alone that this intracortico-ligneous effusion obtains, and, when to this fact we add another, that dicotyledinous plants alone increase diametrically by the successive deposition of concentric layers, it is very likely that the medullary radii are the organs destined for this transverse circulation, and that this subcortical effusion is destined to form these concentric layers.

It has been shewn by Mr. Knight, that the first lymphatic sap, which ascends in Spring, mixes with some of the elaborated sap, which had been formed at the close of the previous season and deposited within the closters during Winter. By wounding a Sycamore tree on a part level with the surface of the earth, sap was obtained which had no particular flavour, and whose specific gravity amounted to 1·004; at the height of seven feet from the ground its weight was increased to 1·008; and, when drawn at a remove of 12 feet from the soil, it weighed 1·012, and had become decidedly sweet. This intermixture is supposed to qualify the lymphatic sap to form buds; a different theory of their formation is given by the author.

The cellular tissue constituting the central medulla of plants appears to Dutrochet to have no connexion with the ascent of the sap, for, by destroying every part of the substance of a branch of the *Rosa Canina*, except the pith, so that no communication could exist between its base and summit but through the central medulla, he found the superior portion of the branch to become dry, to wither and die. Sarrabat and Bonnet likewise observed, that when a cut twig was immersed in coloured liquid none of it was taken up into the pith. Yet, it is certain that the cellular tissue of the medulla in young branches is often full of sap, and Mirbel has endeavoured to demonstrate the existence of pores in the walls of these cells, through which it may pass from one into another. Where the source, or what the use of this central fluid is, has been scarcely more than conjectured. Dupetit-Thauars regarded the pith as the reservoir of a nutritious substance designed for the development of the buds, and into this view Dutrochet partly enters. If a branch of vine, of last year's growth, be examined during Summer while it is covered with buds, the pith will be found of a reddish color and apparently dead, except at the knots which are opposite the buds; and it is certain that it is dry and withered in old branches which wear no buds; while it is spongy and succulent in the young and vegetative. But, in addition to this office, it is maintained by the author to discharge a prior and more important function. In a work, to which we have already had occasion to refer,* he has shewn that the walls of the cells which compose the central pith are covered with globular bodies, which he regards as nervous organs, or *des organes spécialement dépositaires ou producteurs de la force particulière aux êtres vivans, et que l'on nomme force vitale.* These organs vary much in different plants both in size and number, as well as in the same plants at different periods of growth; and the author has observed that the size of the pith bears a tolerably constant and close proportion to the number of these nervous globules, diminishing with their decrease and augmenting with their multiplication. The central system of young branches is almost entirely medulla, enclosed within a medullary sheath. Now, says the author, if we believe that it is during this period of growth that vegetable life is the most active, it is undeniable that this increased activity must depend upon the organs which enter

* *Recherch. Anatom. et Physiolog. sur la Struct. &c.*

into the composition of the new texture, and these organs are the globular corpuscles, which are situated upon the walls of the medullary cells. And if in animals the nervous system be the special seat of life, may we not hold, as analogous to this vital system, those parts of vegetables on which exclusively depends the activity of vital movement, as manifested in the rapidity of vegetable growth? Thus, then, it would appear that the pith, which is a stranger to the ascending sap, receives into its cells such as is elaborated, for the formation of buds and the development of those organs, which are to perform in the economy of vegetable life the functions of nerves.

From these facts and observations we may infer, that the lymphatic tubes are properly the absorbents, or veins of the vegetable system; that the fluid they convey is not destined for vital functions; that the elaborated sap is the vegetable equivalent for arterialized blood in animals; that the closters, whether in the wood or bark, are the channel through which this nutritious fluid filters; that while the crude sap generally ascends and the elaborated sap descends, their movements may be and are frequently inverted; and that while, by the straightness of the lymphatic tubes, together with their destitution both as to branches and valves, it seems the object of Nature to save time and procure velocity, by the ovoid form of the closters, their want of direct communication, and the dependence of all transmission through them on a principle somewhat similar to absorption, it is doubtless that an equally important object is contemplated in the peculiarities of such a mechanism. What this object is it shall be our business to enquire.

This very condensed sketch of the course of the sap in *dicotyledinous* plants is sufficient to prove, that strict analogy will not permit us to dignify it with the term *circulation.* (In *monocotyledinous* plants, its course has not been so fully examined, and is much more imperfectly understood. Their cortical and central systems are exceedingly confused, and many other anatomical difficulties are met with in their investigation, which are not to be encountered in the study of the preceding class; it is highly probable, however, from their general resemblances in lymphatic tubes, closters, tracheæ, and pith, that the same organs have the same uses in both.) The leaves we have seen to be analogous to lungs in animals, the lymphatic tubes to veins, the closters to arteries, the lymph to venous and the elaborated sap to arterial blood; but there is nothing like a central organ through which all the circulating fluid is conveyed, nothing like a continuous series of vessels, nor any thing like a continuous current. The lymphatic sap retrogrades as well as ascends; the elaborated sap returns towards the leaves as well as moves towards the roots; the closters are not continuous tubes, but chains of shut sacs; and the sap often seems to percolate through the general texture of the plant without vascular restraint. While, therefore, in several points strict analogy would be injured by ascribing to the vegetable system the exactitude, harmony and completeness of an unbroken circulation, there are as many features of resemblance between the course of the sap in vegetables and that of the blood in animals, as may entitle the functions carried on by them in both to be considered equivalent, and the machinery employed for their performance tolerable counterparts.

How even this imperfect circulation is conducted, it is not easy to discover; whether by the innate power of the tubes themselves, by corpuscular attraction, by the suction power of the leaves, or any propulsive property in the roots, has been as yet uncertain and unsettled. The author's labours in this department we esteem especially excellent, the results at which he has arrived particularly interesting, and should future investigations verify the principles and confirm the conclusions which he has announced, we shall only be conferring that honor upon merit to which justice lays claim, when Dutrochet shall be enumerated among our most original and successful physiologists. Malpighi ascribed the ascent of the sap to its alternate expansion and dilatation by means of the surrounding atmosphere. Sarrabat explained it upon the principle of the air; which he considered to be contained in the pith and tracheæ, becoming alternately denser and rarer, and thus producing a corresponding ebb and flow in the contents of the sap vessels. Darwin and Hooper accounted for all by irritability. Knight, by the change of volume produced by the action of temperature on the silver grain, which expands during day and collapses during night; and many other theories have been at different times promulgated, into which it would, we fear, be more curious than instructive to enter. The only doctrines which seem to merit a more enlarged notice, before we take up the peculiar views of the author, are three; *the suction power of the leaves, contractility, and capillarity;* and as our main object in examining even these is to shew that neither one nor all can explain the phenomena of vegetable circulation, and that, therefore, some other agent must exist with which we have been hitherto unacquainted, we shall adhere closely to this object, and bear exclusively upon such circumstances as tend to establish it.

When the cut end of a living twig is plunged into water, it is well known that some of the water is absorbed, and that the plant will not only continue fresh, but grow. The quantity of water absorbed by the lower end is, in general, proportioned to the quantity evaporated by the leaves, and the activity, both of the absorption and evaporation, is influenced by the physical state of the surrounding atmosphere. When it is dry and warm, they are increased; when moist and cold, they are diminished; but, whether dry or moist, warm or cold, a very remarkable proportion is maintained between the *outgo* and *income* of the system. Now it is not unreasonable to infer that, since one of these processes, evaporation, is carried on by the leaves, the more abundant the foliage is, the greater will be the quantity of water evaporated; and since both processes, evaporation and absorption, act and re-act so intimately on each other, that one can neither increase nor diminish, without being accompanied by a corresponding variation in the other, it may be inferred that the leaves exert a tolerable influence upon the motion of the sap. These inferences have been already drawn by Hales, and they are both strongly countenanced by the fact, that when the leaves are artificially removed during Summer, or naturally fall in Autumn, vegetation and growth are instantly arrested. As this subject, however, shall come before us hereafter, suffice it, at present, to observe that while we admit the force and application of these facts, and are disposed to attach much importance to the suction-power of the leaves in the circulation of the sap, this power may be found to spring from another and more general agent, without which it would be of small avail; more especially when we know that, in Spring, absorption is commenced and conducted independently of its influence, while the leaves are yet unfolded, and even the buds unformed.

Without advancing any facts to prove that plants, like animals, are endowed with irritability, it may be sufficient to remark, that the phenomena resulting from this principle have led many to maintain that vegetables possess contractile power, and that their fluids are circulated by an agency in every respect similar to that employed in the circulation of the blood. If the stem of a lettuce be cut across, its *suc propre* will be seen to exude from both surfaces—from that belonging to the part which is next the root, and that which is nearest to the leaves; and, as no appreciable difference can be discovered between the velocities with which it pours out of both, it might appear to some that neither the roots nor the leaves are concerned in its motion, since the vessels which open on one surface are attached to no root, and those exposed by the other to no foliage. By what power, therefore, do some ask, can the fluid exude? The following experiment of Van-Marum has been made to answer this query, by ascribing its escape to contractility. This physician, having passed a strong charge of electricity through a stalk of *Euphorbium,* found that the plant poured out no more fluid when cut across, than it would have done had it been simply compressed. Now, reasons Van-Marum, since we know that strong electrical shocks destroy the irritability of animal fibre, we may conclude that they are equally injurious to that of vegetable tissue, and that the reason why the preceding experiment was followed by such a result, is only afforded by such an explanation. The observation of Brugmans and Coulon, had it been verified, would, however, have had a much stronger influence with us; that by means of alum, sulphate of iron, and similar astringents, they could wholly stop vegetable circulation; but Van-Marum, Linck, and Tréviranus, have called this statement in question, and Dutrochet adds to the weight of their contradiction that of his own experience.

"Yet," says our author, "constrained by the necessity of admitting some cause to account for the pressure which fluids in the capillary organs of vegetables evidently sustain, I regarded, as highly probable, the opinion which accounted for the ascent of the sap by vascular contraction, until I was undeceived by the following reflexions. We know that the fig, *Ficus Carica*, contains a large quantity of milky juice in both the cortical and central systems of its young branches. So long as the woody tissue of these branches is in an *herbaceous* state, it is possible that the walls of its vessels may contract upon their contents; but, when this tissue hardens and becomes perfect wood, every idea of contraction, on the part of these vessels, must be abandoned. Now, if we examine the young branches of the fig, towards the end of October, we find the ligneous tissue of their central system indurated to such a degree, that it breaks with noise and cuts with difficulty. The assemblage of tubes and closters, of which it is composed, is so solid, that a quarter of an hour's boiling in nitric acid is necessary to destroy it, yet we see fluids pour out of the rigid tubes of this woody tissue in great plenty, when it is cut or broken transversely. Here I repeat it, it is impossible to admit vascular contractility; and this conclusive fact proves that there is another cause, whatever it may be, to which we must ascribe the pressure to which fluids, contained within the organic cavities of vegetables, experience."

By a very simple experiment, it was first demonstrated by Hales, and afterwards by Mirbel and Chevreul, that the sap in a small vine-branch, retaining its connection with the stem and root, ascends with a momentum sufficient to raise a column of mercury 38 inches high; but if this branch be separated from the plant while the sap is rising, and if its lower extremity be plunged into water, it will be found that the greater part of the ascending momentum is lost, and that, although water will continue to rise in the sap vessels, it will not overflow or reach their extremities. From this single fact it is tolerably certain, that mere capillary power is of itself an insufficient agent, and that the ascent of the sap in plants is a complicated phenomenon, depending on something more active than a *mechanical law*. Capillarity is a power wholly independent of life, the result of certain mechanical principles, and, whether the substance within which it acts be alive, dead, or inanimate, the laws of its operation are unaffected, the degree and duration of its influence the same. How the succeeding phenomena will tally with these observations, the reader will be sufficient to ascertain.

A twig of the *Mercurialis Annua* was separated from the plant, and allowed to wither, until 0·15 of its weight had been lost by evaporation, when it was placed in a vessel full of water, which had been accurately weighed. In a temperature of 12° R. the twig was found to absorb 20½ grains during each of the first four hours, and to evaporate, in the same period, 8½ grains; and although it had resumed its turgid state, its former weight had not been regained. During the next four hours, the temperature and hygrometrical state of the atmosphere being the same, 9½ grains were absorbed, and 9 grains evaporated by the hour; during the night, 7½ grains were absorbed, and 7 grains evaporated by the hour; on the next day, and for some time after, the plant, which was now quite restored to its former freshness, continued to absorb and evaporate between 7 and 8 grains every hour, with this marked and constant variation—that, during night, the absorption was greater than the evaporation, and, during day, the evaporation exceeded the absorption.

Another branch from the same plant was withered, until 0·36 of its primitive weight had been lost, when it was placed in a vessel with water, under circumstances precisely similar to those followed in the preceding case. During the first 24 hours it regained only 12 grs. of its weight, or, in other words, 12 grs. were the excess of its absorption over its evaporation; on the following day it absorbed one grain and a half only every hour, and, as it lost by evaporation more than it took up, it soon began to dry, and in a few days, except two of its lower branches, died. In this experiment it appears that the vitality of the plant was too much injured during the process of desiccation to conduct an unimpaired circulation, and that while, perhaps, by capillarity death was delayed for a period, the motion of the sap was not so perfectly sustained as to uphold life. But the succeeding experiment renders it very questionable, whether, even in this last case, the protraction of life depended on the action of capillarity.

Two other branches of a *Mercurialis* were taken; one was made to lose by evaporation 0·61 of its weight, and the other 0·72. The first had many of its leaves in a state of *souplesse*, but those of the second were more dried, and even crepitated upon handling. These branches were completely immersed in water, and some twigs of the first branch revived, became fresh and turgid; but no appearances of vitality returned to the other. They were then taken out of the vessel in which they were immersed, and had their extremities placed in a foot of water. The twigs of the first branch, which had revived during the previous immersion, continued fresh and active, but none of the others were recovered; and the second plant continued dead and flaccid.

How can such experiments, which were variously modified yet produced the same results, be explained by capillary principle? Why should a mere mechanical and dead power be so controlled by vitality, as even to cease to act upon the extinction of life, or, at least, to act so efficiently as to prevent death? and why should the physical conditions of the atmosphere so materially modify its results? It has been proved by Hales that light also has much influence upon the functional actions of vegetables; that plants transpire much more in the light than in the shade; and we have already seen that the ratio, which obtains between evaporation and absorption during day, is reversed during night. The application of this last phenomenon to the present point might be disputed, were it produced by the direct rays of the sun only, which, by acting on the leaves, would increase evaporation; but it is equally occasioned by diffuse light, which is not supposed to exert any such influence over these respiratory organs. Many other arguments might be added; but, as they will suggest themselves to the reader as we advance in our explanation of the laws of a new principle, which it will be now our business to unfold, we shall not dwell upon this point any longer.

The conclusion to be drawn from all these observations unquestionably is, that neither the suction-power of the leaves, nor the contractility nor capillarity of the sap vessels, can explain all the phenomena of vegetable circulation, and that, while something may be done by each and more by all of them, something shall still remain undone, which we have hitherto had no known agent to perform. To fill up this *hiatus* in our physiology of this function has Dutrochet come forward, and we shall now endeavour, in the first place, to review the evidence on which the existence and agency of this principle are founded, to develop its nature and explain its laws, and then conclude by applying it to the illustration of such phenomena, both vegetable and animal, as are inexplicable by any or all of the preceding doctrines, and seem to come within the limits of its influence.

In consequence of appearances, which were observed during the vegetation of some aquatic plants and the copulation of snails, the author was led to the conclusion that water, by accumulating within small organic cavities, endows these cavities with a propelling power, whereby their contents are expelled; and that such accumulation always occurs in such cavities, when their contents are of a denser consistence than water. "Such, then," says the author, "are the two facts, upon the observation of which I have established the existence of a new physicoorganic action, which possesses the two-fold property of rendering turgid small organic cavities, and of expelling the fluids which such cavities contain. A double effect which has its origin in a single cause, in an unceasing accumulation, *et avec excès*, of water within these organs. As this new physico-organic principle requires to be designated by a new name, I shall distinguish by the term *Endosmose** the agent, in virtue of which small hollow organs are filled with a fluid, which seems to enter their cavities with force. The existence of this vital principle, being thus established by the observation of spontaneous operations in organic nature, we shall now endeavour to confirm by experiment."

From witnessing the turgidity acquired by the spermatic sacs of the snail, the author imagined that an analogous result might be obtained by introducing into the intestines of small animals, before immersing them in water, an organic fluid of greater density than the liquid which surrounded it. With this view the cæcum of a pullet, after having been thoroughly cleansed by injections of pure water, was half filled with milk, tied at both ends, and plunged into a vessel containing rain water, whose temperature was between 18° and 21° R. Before immersion it weighed 196 grains, in 24 hours afterwards 269 grains, and at the lapse of 36 hours from the commencement of the experiment, the water having been renewed night and morning, its weight amounted to 313 grains, or 117 grains of water had been absorbed into the interior of the gut, which had become in consequence much distended. From this time the intestine grew lighter, its turgidity was lost, and in 36 hours it weighed only 259 grains. The ligatures were then removed, and on opening the gut the milk was found curdled and putrid.

The same portion was again well cleansed, half filled with fresh milk, and replaced in water. After 24 hours' immersion 21 grains were gained, but from this period absorption ceased, and in 24 hours after, on removing the ligature, the milk was found in a similar condition as at the close of the preceding experiment.

A second portion of cæcum, which was half filled with rain water, weighed 64 grains, and was then immersed in water at the temperature of 24° R. At the close of 36 hours it weighed 121 grains, having absorbed 57; but from this period it began to empty, and after three hours the enclosed water was found putrid. The gut was now emptied, cleansed, again charged with fresh water, and replaced in water for 36 hours. Some absorption occurred; but at the expiration of this time the water was found putrid. The weight of the cæcum before immersion was 63 grains, and during it seven only had been acquired. After again removing the putrid water and purifying the gut, as much milk was poured into it as made it weigh 66 grains, when it was again immersed in water. In 36 hours 58 grains were added to the original weight, but immediately after decrease commenced, when the gut was opened, and the milk found curdled. The pieces of gut used in all these expriments had become somewhat putrid.

A third portion of cæcum belonging to a pullet was well washed, then carefully emptied, tied up at both ends, and plunged into water. Before immersion it weighed 27 grains, and, after it had remained 12 hours steeping, 13 grains of water were found in its interior.

A fourth piece of a similar gut was half filled with albumen and plunged into rain water, of the temperature 18°—21° R. Eight hours and a half afterwards the cæcum, which at first weighed 58 grains, had become weightier by 72 grains, and was very turgid. This turgidity continued for three days, and the gut, which was nearly as full as it could be made, gained three grains only during all that time. On the beginning of the fourth day it began to lose weight, and on the fifth it was lighter by 22 grains. The gut was then untied, emptied of a putrid, flocculent fluid, into which the albumen had degenerated, was well

* Derived from two Greek words—*ἔνδον*, within, and *ωσμος*, impulse.

washed, and again charged with the same kind of substance. When the gut was immersed it weighed 60 grains, eight hours afterwards 92, twelve hours after it was five grains lighter, and this decrease increased during two days, until the total loss amounted to 20 grains, when it was opened, the putrid albumen removed, and a solution of gum substituted. In this state the cæcum weighed 48 grains, and five hours afterwards 124 grains, when it became turgid. Its turgidity continued for two days, on the third loss of weight was manifested, and on the fourth it was *minus* 48 grains; when the cæcum was opened, its contents were found putrid, and the gut itself decomposing.

These experiments we adduce as a *fac simile* merely of many very interesting and variously modified trials, which it would be unnecessary to occupy our time and pages by multiplying, as all the results are strikingly similar, and as the above, we presume, will be sufficient to show that by the agency of a certain principle, with whose laws we are not as yet acquainted, fluids in a healthy state can be drawn through an organic membrane with surprising velocity, and that although, as we shall see more largely hereafter, the presence of a denser fluid within gives an increased velocity to the entrance of a rarer fluid without, the endosmosmic principle can act when both fluids possess equal density, and even when there is no enclosed fluid of any sort. Now we believe that the results of these experiments can receive no explanation from capillary power, for capillarity acts upon putrid as well as upon sound fluids, through rotten as well as through sound membranes, uninfluenced by the density of the former, or the soundness of the latter. Neither can they be accounted for by the structure of the membranes employed, since experiments were made with an inverted cæcum, and with one deprived of its mucous coat, without the occurrence of any appreciable variation. Nor, lastly, can any affinity, existing between the rarer and denser fluids, solve the problem, because, by a subsequent experiment in a graduated glass tube, into the lower half of which was poured an albuminous solution, and into the upper half pure water, so that these two liquids were placed in actual contact without being allowed to intermix, no absorption of the one by the other was found to have occurred after the lapse of 48 hours, as the line of demarcation between the two fluids stood at the same degree, by which it was noted before the experiment commenced.

It is remarkable in the above experiments, that so soon as the contents of the gut began to putrify, not only did the outer fluid cease to enter, but the inner fluid began to exude. This *sortie* was at first ascribed by the author to the pressure of the walls of the gut upon its contents, after the cessation of the endosmose; but the rapidity of this exudation was frequently so marked, that it was ultimately suspected to be the result of an action opposite in direction to endosmose, but similar in cause, and the succeeding experiment confirmed this suspicion. Three-fourths of the cæcum of a pullet were filled with water, which held in solution one-fiftieth of its weight of gum arabic, after which it was tied at both ends, and plunged into pure water. At the end of an hour it had gained six grains, when the pure water, in which it was immersed, was changed for water holding in solution one-tenth of its weight of gum arabic. At the expiration of an hour it had lost 30 grains, and after the lapse of two hours the gut had become nearly empty. It is evident, therefore, that whether the denser fluid be interior or exterior, it always forms the centre of attraction to the rarer fluid, and that, while the direction of the current thus formed may be reversed, the moving principle is the same in both. The author has, however, distinguished by a different name this inverted action, and while inward attraction is called *Endosmose*, outward impulse is called *Exosmose.** By virtue of this principle he conceives the fluids employed in the foregoing experiments not only ceased to excite endosmose, so soon as putrefaction began, but commenced an outward

* Derived from εξ, aut, and ωσμος, impulse.

movement. This explanation, however, is scarcely satisfactory, for it would require to be shown, that the putrid solutions of the albumen and the gum had become rarer than the external fluid, before exosmose can furnish any explanation. Might not the chemical alterations which putrefaction had established modify the functions of these organs? This is rendered more than probable by the succeeding results. Rain water, with a very small admixture of Sal Ammoniac, was enclosed within the cæcum of a pullet, and immersed in pure water. About an hour afterwards it was weighed, and 20 grains were found to have been absorbed. This experiment was modified by reversing the situation of the fluids, and the effect was reversed, or weight was lost. Now, since a solution of Sal Ammoniac is rarer than pure water, when such solution was made the included fluid, exosmose should have been excited, and when the surrounding fluid, endosmose; yet the very contrary occurred, shewing clearly that it is not difference of density alone which excites these powers, but that they also result from difference in chemical constitution; and it is very remarkable that alkalies and acids have exactly opposite influences upon these physico-organic principles, the former producing endosmose, the latter exosmose. One exception to this general rule is met with in Sulphuric Acid, and this curious fact is another proof, that greater density in the included fluid is neither the *sine qua non* of endosmose, nor, in all instances, sufficient to produce it. To prove this, a very simple instrument, which Dutrochet calls an *Endosmometer*, was used. This instrument is nothing more than a glass tube having a small bore, to one end of which is tied a thin piece of membrane, as bladder, and to which is attached a graduated scale. To prevent this membrane, by becoming flaccid through maceration, from giving rise to any error in the results, a cribriform plate or floor is fixed underneath it, upon which the membrane may rest. Now into this instrument some sulphuric acid was poured, and then the end of the endosmometer was immersed in water. Immediately the acid began to fall in the tube, shewing that exosmose was operating, and that, in place of a denser fluid producing absorption, exudation was occasioned. This exudation seems, however, to arise from gravity, and not from exosmose, for if some sulphuric acid be poured into a solution of gum, which is highly productive of endosmose, the endosmose ceases, but no exosmose is produced.

This fact has formed the groundwork of a division of fluids, with reference to endosmose, into *active* and *inactive*. Among the *active*, are animal and vegetable liquids in a sound state, as solutions of gelatine, albumen, and extractive, solutions of gums and sugar, and all emulsions; these Dutrochet designates *organic liquids;* all saline and alkaline solutions, alcohol and acids, with the exception of the sulphuric; these are called *chemical liquids*. By all of these, endosmose is energetically elicited, (under the term endosmose, we here include exosmose, of course) but more especially by the *organic* class, for the *chemicals* are endowed with the singular peculiarity of ultimately destroying the action which they at first establish. Thus, if some alcohol be added to a solution of sugar, the endosmosmic power will be at first increased, but, in the course of some hours, entirely destroyed. Among the *inactive* fluids, the most remarkable are putrid liquids and sulphuric acid. A very ingenious suite of experiments have rendered it tolerably certain, that the endosmosmic incapacity of this class seems to arise from the presence of sulphuretted hydrogen. Having poured into the reservoir of the endosmometer some water, charged with 0·05 of its weight of gum, and having plunged it into water containing some hydrosulphuret of ammonia in solution, endosmose manifested itself for four hours, after which it ceased, and the fluid descended in the tube. The instrument was then emptied, and being well washed, was again charged with a similar solution of gum, and again immersed in hydrosulphuretted water, but no endosmose was produced, and the liquid sunk gradually in the tube. Some feculent matter was now taken from the large intestines of a pullet, was mixed with its own weight of water, and then introduced into the reservoir which was plunged into water. A slight ascent was at first perceptible, but it did not continue long, and, although the densities of the included and surrounding fluids bore no proportion to each other, the fæces fell in the tube, or exosmose was produced. Equal parts of this feculent matter, and of a solution of gum arabic, were mixed together, and put into the endosmometer, but it rapidly fell in the tube ; the quantity of gum was increased, yet endosmose was not excited, and it was not until 0·2 of the weight of the solution consisted of gum, that any inward impulse was induced. It is, therefore, to the sulphuretted hydrogen which it contains, that feculent matter owes its endosmosmic incapacity, and it is, probably, to the same cause we are to attribute the inactivity of putrid animal liquids, since sulphuretted hydrogen is evolved during animal putrefaction. From all these observations and experiments, it is obvious that all liquids, which exert any influence over this principle, may be arranged into three classes ;—1st, Such as furnish a constant supply of endosmose, until the fluids, or membranes used, become unfit for the action of the principle ; 2d, Such as possess the twofold property of producing and destroying endosmose ; and, 3d, Such as are *sedatives* of endosmose, of which, as yet, two only are known—sulphuric acid and sulphuretted hydrogen.

So far as we have yet seen, endosmose and exosmose seem to act separately, the one commencing on the cessation of the other, but, in reality, they are simultaneously operating agents ; while the rarer fluid is carried in excess to that which is denser, the denser fluid passes to that which is rarer, and thus two opposite and unequal, yet not opposing, currents are carried on across one and the same membrane. A solution of the hydrochlorate of soda was enclosed in the endosmometer and surrounded with water, and endosmose was shortly indicated by the ascent of the solution in the tube. At the same time, the presence of the solution in the exterior fluid was detected, at first by nitrate of silver, and afterwards by the taste, proving that endosmose and exosmose operated simultaneously, while the action of the former evidently predominated. In the same manner were tried various salts, and even organic liquids gave the same results. A solution of gum, coloured with indigo, was put into the endosmometer and immersed in water. In a short time the action of endosmose was apparent, and the blue color, which the surrounding water gradually assumed, shewed that some of the indigo had escaped through the membrane. This *descending filtration* is not the effect of gravity, for when alcohol, or any liquid lighter than water, is inclosed in the tube, the operation of exosmose is as evident as that of endosmose, although inferior in degree ; so that the accumulation of a liquid on one side or other of an organic membrane, is only the amount of the excess of one current over its opposite. We specify an *organic* membrane, because these vital principles are almost exclusively confined to vegetable and animal fibre. To discover whether endosmose would operate through a *mineral medium*, very thin laminæ of white marble, fine sand stone, crystallized sulphate of lime, and slate, were substituted for the piece of bladder which was tied to the end of the endosmometer. A solution of gum arabic was then introduced into the reservoir, and it was immersed in pure water; but no ascent of the fluid could be observed. Baked argil was the only mineral substance which excited endosmose, and it was found that, when a very thin lamina was employed, and a solution of gum arabic was the fluid used, there was very little difference between the activity of the absorption occasioned, and that manifested when an organic membrane was the medium. But when a few drops of sulphuric acid were mixed with the solution of gum, endosmose was arrested, shewing that this mineral was subject to the very same laws with organic membranes.

It, therefore, appears, that of all mineral solids hitherto tried, none can excite endosmose but such as are aluminous, and that we may divide *solids*, as well as *fluids*, into *inactive* and *active*, or into those which elicit endosmose and those which do not.

The *velocity* of movement occasioned by endosmose is generally proportional to the excess of the density of the interior fluid over that which is exterior. A

solution of 1 part of sugar and 3 parts of water, the density of which was 1·083, was enclosed within the endosmometer, which was plunged into rain water, and 19° of ascent were obtained at the close of an hour and a half. A solution of 2 parts of sugar and 4 parts of water, density 1·145, was introduced into the same instrument, and 34° were obtained after the same period. A third solution of 4 parts of sugar and 4 parts of water, density 1·228, gave 53° at the end of the same time, and by the same instrument. For the purpose of measuring the *force* of endosmose Dutrochet constructed an apparatus somewhat similar to that employed by Hales, Mirbel and Chevreul, to ascertain the ascensional force of the sap in the vine. It is merely a common endosmometer, the tube of which, in place of being straight, curves twice upon itself; but for a more minute description we must refer to the original, where the reader will be furnished with a plate. Into the reservoir of this instrument a solution of sugar, of the density of 1·025, was enclosed, and in 28 hours it had raised an inch of mercury in the tube 10 inches and 7 lines; a second solution, density 1·053, was tried, and in 36 hours it had raised 10 inches of mercury 22 inches and 10 lines; a third solution, density 1·110, elevated 22 inches, in two days, 45 inches and 9 lines. These three experiments were made in an apartment whose temperature was 16½° R, and it is obvious from their results that the law, which we have above announced as applying to the *velocity* of endosmose, will equally apply to its power;—that is to say, that the force of endosmose is proportional to the excess of the density of the interior over the exterior liquid. The saccharine solutions employed in the above experiments were of different densities, expressed by 1·025, 1·053, and 1·110; so that the excess of the density of these solutions over the exterior fluid, which was water, its density being regarded as 1·000, was 0·025, 0·053, 0·110; and if we establish a similar progression of effects, by chusing for our first term 10 inches and 7 lines,—the height to which the column of mercury was raised by the first solution,—we shall have 10 inches and 7 lines; 22 inches 10 lines; 45 inches 9 lines. The existence and operation of this law being thus indisputably established, a knowledge of the density of the fluids used is sufficient to enable us to discover the amount of endosmosmic effect which they can produce, and a knowledge of the effects produced is sufficient to enable us to ascertain the density of the fluids used; or, in other words, the density of the fluids being given the amount of endosmose may be found, and the amount of endosmose being given the density of the fluids may be found. So that by means of a common endosmometer, such as was employed in these experiments, the temperature being always the same, a syrup made of sugar, of the density 1·3, would generate such an endosmosmic force as could raise a weight equal to four atmospheres and a half!

Dutrochet believes that the seat of endosmose is in the capillary pores of the *active* partition which separates the two heterogeneous fluids. But we have seen that *active* fluids are as necessary to its existence as *active* solids, that when sulphuric acid was poured into the receiver of the endosmometer the membrane was converted from an active to an inactive medium, and that, until the incapacitating influence of this *sedative* was removed by ablution, no endosmose was manifested. When an active fluid was brought into contact with an inactive medium no endosmose was elicited; when an active medium was exposed to an inactive fluid the same result occurred; when both medium and fluid were active endosmose was produced; and when both were inactive none was generated. An inactive fluid renders a medium naturally active inactive, and an inactive medium makes a fluid of itself active perfectly inert; so that we can see no superiority on the part of the medium over the liquid, nor of the liquid over the medium, and the author's reasoning on the nature of inflammation, which we shall adduce hereafter, would countenance a view the very opposite. We do, therefore, imagine that active fluids being thus as essential as active solids to the operation of endosmose, it is more philosophical in the present state of the subject to say that this principle results from the contact and co-operation of both, than that it has its seat exclusively in either.

What the essence of this mysterious principle is has not as yet been clearly revealed, but experiments have gone far in tracing it to *electricity*. M. Becquerel has proved that the contact of liquids with solids excites electrical phenomena, when the liquids are such as may act upon the solids chemically. M. Nollet has ascertained that electricity accelerates the movements of liquids, and the following curious experiment in the Annals de Chimie has placed it beyond a doubt that galvanism has the property of communicating to them a very considerable impulse. Having separated a vessel into two compartments by a partition of bladder, filled one of them with water, and put a few drops only into the other, the positive pole of a galvanic pile was placed in the full apartment, and the negative pole into that which was nearly empty. An active current was immediately established across the membranous partition, the water was made to exchange cavities, and ultimately rose to a height in what had been the empty division, even greater than that at which it stood in the division which had been at first full. Now, argues Dutrochet, the zinc employed in the galvanic pile is equivalent to the rarer, and the copper to the denser fluids, and as the negative pole is represented by the former, and the positive pole by the latter, the course of the water in the above experiment exactly corresponds with that followed during the generation of endosmose;—or, in other words, it is directed from the rarer to the denser body. This similarity is too striking to lead to no suspicion of the identity of endosmosmic and electrical agency, and the supposition which it encourages meets with still stronger support from the following phenomena.

The cæcum of a pullet was tied to the extremity of a glass tube, and the opening in the end of this tube was closed by a piece of cork, through the centre of which a copper wire was passed from the tube into the cæcum. This wire was intended to establish a communication between the interior of the gut and the negative pole of the pile, and the glass tube served to insulate the water which was in communication with the positive pole from that in which the cæcum was plunged. The cæcum thus situated was an empty sac, into which nothing could enter but through its coats. The action of a small pile was now applied, and in twenty minutes the gut was filled to turgidity. The relation of the poles was now reversed, and the positive end of the pile made to correspond with the interior of the gut, but not a drop of water was introduced. In order to prevent the accumulation of hydrogen by the decomposition of the water, as some had been generated in the last experiment, the wire was introduced into a very fine glass tube open at both ends, and both were passed through the cork into the interior of the cæcum, so that the gas might be removed as soon as it was evolved, and the cavity of the gut left free to the admission of water. The pile was now applied, the gut immediately began to fill, and quickly became turgid; in half an hour the water rose in the capillary tube, poured over its superior end, and only ceased to flow when the action of the pile became weak. It is known that when a salt, having an alkaline base, is subjected to the action of galvanism, it is decomposed, the alkali is deposited at the negative, and the acid at the positive pole. Now, since the negative end of the battery corresponds with a rarer, and the positive with a denser fluid, this disposition of the constituents of the salt is in strict accordance with the laws of endosmose.

But difference of density in the employed fluids is not necessary for the production of endosmose by galvanism. Some distilled water was put into the reservoir, and the endosmometer was plunged into distilled water; the wire, which was connected with the negative pole of the pile, was then brought into contact with the interior water, and that belonging to the positive pole with the exterior water. Immediately ascent was perceptible in the tube, the water soon overflowed its upper end, and the current ceased only with the energy of the battery. The phenomena of endosmose having been thus generated by galvanism, a series of similar experiments were commenced to discover whether exosmose could also be elicited. The cæcum of a pullet was filled with water and every thing about the apparatus was similarly arranged as in the preceding experiment, except that the situations of the poles were reversed, the positive wire being placed in the interior water. An electrical current, directed from the positive to the negative pole, drove the water which was enclosed in the gut through its coats, and in less than half an hour left it entirely empty. A pod of the *Colutea Arborescens* was substituted for the animal membrane, and the same results were obtained.

It is certain, therefore, that both endosmose and exosmose can be elicited by galvanic electricity, and the alternative of two inferences is unavoidable;—either that these agents depend upon galvanic electricity for their activity, or that they can be generated by galvanism, as well as by the contact of active liquids with an active medium. Had we space the analogy between these powers might still be more closely traced; for it has been ascertained by repeated experiments that all solids, which are active in eliciting endosmose by the co-operation of active fluids, are likewise active under the influence of electricity;—that the reverse of this fact is equally true;—that, with the exception of sulphuretted hydrogen, which seemed to have no sedative effect upon the generation of endosmose by electricity, the active and inactive fluids stood in the same relation to it with the active and inactive solids;—that endosmose and exosmose are simultaneously operating principles, whether elicited by the pile, or by the agency of active solids and fluids;—that elevation of temperature in the zinc and copper is accompanied by increase of action in the pile, a fact which also obtains in endosmose commonly produced;—and, finally, it has been found that when the cæcum of a pullet has been nearly filled with white of egg and introduced into water, it soon becomes turgid from the operation of endosmose, and when it is opened some hours after the experiment, its interior surface is found coated with a membrane formed of coagulated albumen; but, while it is well known that albumen is coagulated by electricity, no cause for such an effect in this case can be discovered in any part of the apparatus employed in the experiment.

Having now at sufficient length, and, we hope with sufficient perspicuity, established the existence of a physico-organic agent of great power, whose mode of operation has not been hitherto understood; having detailed its phenomena and illustrated its habitudes; having examined its essence and ascertained its seat; having investigated the conditions of its agency and calculated the amount of its power; it only remains to apply it to such departments in animal and vegetable life as it may serve to illustrate; and it is presumed that the facts, which have been advanced during this inquiry, are quite equal to prove that if such an agent can be made to operate, without any diminution of its momentum or any modification of its laws, upon living beings and within moving mechanism, it will be amply capable of solving difficulties hitherto unexplained, and of executing functions, whose motive powers and machinery were almost equally unknown. That such an agent does operate on living beings and in living mechanism, we shall now enter into the author's reasoning to prove;—and, first, with respect to vegetables.

The vesicles, or cells of which vegetables are composed, generally contain organic liquids denser than water. To prove this would require more time than we have now to spare; but let him, who scruples to admit this assumption, look into the *Recherch. Anat. &c.* of Dutrochet, and he will therein be furnished with the required evidence. These cells, consequently, ought, in accordance with the laws of endosmose, to generate this agent immediately their exterior walls are brought into contact with water. Turgidity of these cells is then the next and necessary consequence; but we have previously shown that turgidity is inseparably connected with and essential to motion, so that it is only requisite to suppose that these cells are externally exposed to water, to have an agency generated which shall be sufficient to explain all the phenomena of vegetable circulation. In addition to these facts, many other conditions of endosmose will be found in vegetables. Thus, it has been proved that, so soon as plants begin to spoil, or, in other words, so soon as the fluids included in their cells become putrescent, the turgid state is lost on which depends the ascent of the sap; but endosmose ceases when the denser fluid becomes putrid, therefore endosmose and this turgid state are either intimately, or inseparably connected. It has likewise been proved, that increased temperature is a *stimulus* of endosmose, and the influence of temperature upon vegetable growth is universally admitted. Each vesicle or cell absorbs and exhales continually, and the sum of the fluid gained, or lost, is only the excess of one of these actions over the other; but so is it with these physico-organic principles, they operate simultaneously, and the existence of endosmose, or of exosmose, is merely the result of the predominancy of one of them. These points of analogy are given only as a specimen of such as might be advanced in proof of the position, that endosmose and exosmose operate on living vegetable fibre, and that both the afflux of the sap to the roots and its impulse along the vessels can be most satisfactorily accounted for by the agency of these principles alone.

To explain the mechanism, by which this afflux and impulse are carried on, it is necessary to observe that there are small cellular organs at the end of the roots, called by Decandolle *spongiolæ*, (because even by him they were believed to have an absorbing power, although this power was not properly understood,) which are the grand seat of impulse in all vegetables; and, though they imbibe as well as impel, there can be no doubt that the latter action is the stronger, and that their principal function is to send upwards to the leaves the lymphatic sap. If the upper part of a branch of vine be cut across during Spring, the sap will pour over the incised surface; if the stem be cut across during this discharge, the ascent of the sap in the separated part is immediately arrested, and none is found to overflow; but if the cut surface of that portion of the stem, which continues attached to the soil, be examined, the sap will be seen to exude from it as actively as it did from the upper surface before the lower cut was made; and similar amputations, made from above downwards even along the roots, will be followed with similar results, until we arrive at these cellular cones, or spongiolæ, when all circulation will be effectually destroyed by their removal. From this simple experiment, it is very obvious that the sap tubes have very little, if any share in the circulation of their contents, and that it is to the spongiolæ we must look for, at least, one cause of their ascent. These bodies, when situated under ground, are always surrounded with water, the cells which compose their interior structure contain a fluid denser than water, and the result of such physical conditions must, therefore, be endosmose; for we have not only two active fluids and an active medium, but the internal fluid is the denser of the two. The endosmose thus generated fills these spongiolæ, and, since nothing introduced within them can evaporate as in the leaves, that turgid state is produced upon which impulse depends. But the lymphatic tubes are the *excretory ducts* of these glands, so that when the impulse begins, the contents of the spongiolæ ascend in the sap vessels, more water is then imbibed to supply the place of what has just been expelled, a fresh impulse is the consequence of a fresh state of turgidity, the ascending column of sap is urged along by this *vis a tergo*, and thus is established an ascensional movement, which Mirbel and Chevreul have found to be supported by a pressure superior to that of the atmosphere.

We have said that the lymphatic tubes do not appear to take an active part in the ascent of the sap, and our assertion has been grounded upon a very simple experiment. But it may be asked, if this be true, how do branches, which have been separated from their roots, continue to vegetate, when their cut extremities are immersed in water? A branch of *Mercurialis* was placed in water, to which one-forty-eighth of its weight of concentrated sulphuric acid was added. At the end of four days the acid had risen in the plant above the level of the fluid in which it was immersed, and although every portion of the plant, except the leaves, had been killed by the acid and had become brown, absorption continued.

In this case the acid had not reached the leaves and, therefore, they escaped, but having penetrated every other part, every other part was rendered incapable of contracting, because deprived of life; but absorption was carried on till the very last, so that both afflux and impulse must have depended on the suction-power of the leaves. Now this suction-power is only another name for endosmose, because, constituted like the spongiolæ, leaves both exhale and inhale moisture. The leaves and spongiolæ are, then, to be considered the *heart* and *lungs* of the circulation of the sap in vegetables. By one of these organs it is drawn up, by the other it is propelled, by both it rises. But they are more than heart and lungs; they are also *absorbents*. The spongiolæ attract as well as expel, the leaves expel as well as attract; the spongiolæ cannot expel until they have attracted, the leaves cannot attract until they have expelled. They are in fact possessed of the same powers inverted. Attraction is predominant in the one; impulse in the other. The leaves are the origin of a feeble impulse, and the centre of a strong attraction; while the spongiolæ are the centre of a feeble attraction, and the origin of a strong impulse. So that, in the strictest sense of the expression, both spongiolæ and leaves are *double self-working pumps*, made to fill and empty themselves by the same machinery and at the same moment. The endosmosmic power of the spongiolæ occasions an afflux of the fluids which surround the roots, this afflux keeps these organs in a constant state of turgidity, this turgidity generates an impulsive power, this impulsive power acting on the enclosed fluid raises it in the sap vessels, this ascent is unceasingly supported by the unceasing introduction of fresh fluid into the spongiolæ, and when the weight of the ascending column becomes unfavourable to its progress as it approaches the leaves, the suction-power of these organs adds its influence to the *vis a tergo* of the roots, and the motion which exosmose commenced endosmose completes. The crude and indigested sap is now converted into an elaborated and nutritious fluid; the leaves which strongly attract weakly impel; the liquid which ascended in the lymphatic tubes now begins to descend in the closters of the bark and wood, which we have shown to be endosmosmic organs; and the moving powers of the leaves and closters must be somewhat assisted by the force of gravity.

The ascent of the sap by impulse is complete only in the lymphatic tubes whose caliber is continuous; in the closters of the wood and bark, in the stems of grasses which are studded with knots, and in the vessels of young plants which are interrupted by numerous partitions, its motion is mainly conducted by afflux, exerted by one organic cavity upon its neighbour. At first sight this difference of structure might appear unfavourable to motion, but if the laws of the agents, which we have endeavoured to unfold, be properly undertood, it will be evident that the very reverse is the truth. Each of these closters and each division of these partitioned tubes is an endosmosmic organ, or an *organic leyden vial*, and is, consequently, possessed of powers within itself sufficient to execute the transmission of its own fluids; and when the separate actions of all these centres of motion are united, the sum of power must equal, if not exceed, the impulse communicated by the spongiolæ of the roots. The action of these closters must also be kept in view in tracing the progress of the sap through the central medulla, the medullary radii, and every other portion of the vegetable structure, seeing by it alone we can account for such partial and irregular movements.

As the sap thus makes its way, by the operation of endosmose, into every fibre and every cell, as every fibre and cell is endowed with endosmose, and as endosmose never operates alone, but simultaneously with exosmose, almost every phenomenon of vegetable life, as well as the circulation of the sap, can be traced to the agency of these two physico-organic principles. To them are we to ascribe secretion and excretion, inhalation and exhalation, composition and decomposition, the ascent of the stem and the descent of the roots, irritability and contractility. But, as it is quite impossible for us to enter at present upon so extensive a field, we shall confine ourselves to one or two observations on some of their most remarkable operations. By a great many experiments and observations made upon the *Impatiens Balsamina, Momordica Elaterium, Mimosa Pudica,* and other plants, Dutrochet concludes that the irritability of the ovary of the first, of the fruit of the second, and of the leaves of the last, consists of incurvability and contractile power. This incurvability depends on the decreasing size of the vesicles which compose the irritable tissue; these vesicles contain an organic fluid denser than water; when they are exposed to a rarer liquid they exercise endosmose, and the irritable tissue so bends that the largest vesicles occupy the convex side; when they are exposed to a denser liquid, they produce exosmose, and two effects follow;—the irritable tissue so bends that the smallest vesicles are on the convex side, and at the same time it contracts, or shortens, by the partial evacuation of the fluids they contain. The cells of plants are ever changing their fluids, and the fluids are ever changing their properties; in this way is alburnum converted into duramen, and each secretion formed.

It must be now tolerably evident to the attentive reader, that capillarity has little to do in any of these actions. Will capillarity absorb one fluid and reject another; will it *expel* as well as imbibe; will it act vigorously with sound fluids and slowly with such as spoil; will it absorb through one medium and not another, although the porosity of both be equal; will it support two currents at the same time, causing the stronger current to move in the direction of the denser fluid; in fine, will an agent, whose effects are limited to so contracted a sphere, explain the diversified, numerous, and brilliant operations of vegetable life? It is certainly through capillary pores that endosmose operates, and not through vessels made exclusively, and exclusively devoted to that purpose; but we know nothing either of their number, shape, or size; and it is only in this single point that capillarity and endosmose can be found alike.

We have been thus minute upon the application of these principles to vegetable life, because so many points exist in common between the endosmosmic properties of plants and animals, that a full explication of the one will save much repetition during our investigation of the other. There is one marked peculiarity, however, between these two classes of beings;—the contractility, which is possessed by animals in a high degree, we have seen to be exceedingly weak in vegetables, and, therefore, many of the functions of animal life are exclusively performed by this *extra* property. While the blood, for example, continues in the arteries, its motion is almost, if not altogether, owing to the impulse of the heart and the contractions of the vessels; the phenomena of muscularity and locomotion are dependent on the same power. It is not unlikely that this contractility has a close connexion with endosmose in animal as well as vegetable fibre; but at present we shall only follow the author, while he traces its operations in the minute structure and functions of the animal system. It has been already frequently observed that the fundamental conditions of endosmose are vesicular structure and the existence of a dense organic fluid in these vesicles. These conditions have been found in vegetables, in animals they equally obtain. In the *molluscæ* the vesicular texture is very evident; even more so than in vertebrated animals. With a good microscope the walls of the vesicles, in certain parts of these creatures, may be seen to consist of an aggregation of smaller ones, but all, both large and small, are filled with organic matter. Among these vesicles blood-vessels ramify, but no immediate communication obtaining between them, the fluid they introduce must be transmitted through their capillary pores by filtration, which, as we have already seen in vegetables, is another name for endosmose. In *inflammation* the soft parts become turgid, the fluids in the neighbourhood of this turgescence are attracted towards it, an increased velocity is communicated to the local circulation, and thus is there formed a new or morbid centre of afflux and origin of impulse, or, in other words, endosmose is generated. Now this principle, whose effects are so visible in disease, likewise exists in a normal state, but in an inferior degree. The capillary circulation is conducted by it. The blood of the small arteries is drawn into their capillary branches, and it is to this afflux that the empty condition of arteries after death is to be ascribed; the blood of the veins receives in their capillary ramifications an impulse arising out of the previous afflux, which is analogous to that impressed upon the sap in vegetables by the leaves, and when there are no capillary branches between the arterial and venous trunks, the impulse of the heart extends along the entire vessel, and alone conducts the circulation of its contents. In the *Salamander*, for instance, we see only a single blood-vessel, which, when it has arrived at the extremity of the tail, bends and returns to the heart, without presenting any distinction of artery and vein. Indeed, Spallanzani imagined that in all reptiles the action of the heart was the sole moving power in the circulation, and this opinion may be true so long as there are few capillaries, but when the arteries ramify minutely the force of the heart is lost in the capillary circulation, and the blood proceeds under the influence of a new power. It is popular to believe that the smallest blood-vessels contract, but were this so, a dyastole and systole were as necessary for them as for the heart, yet it is impossible to detect the slightest movement towards contraction or dilatation in their walls. The circulation of the blood, therefore, is a complex phenomenon, occasioned by the co-operation of many very different causes, but among the principal must be ranked contraction of the heart at one end and endosmose of the capillaries at the other.

These remarks will equally apply to the lymphatic and lacteal systems, but as no sufficient cause has been yet discovered to solve the problem of their functions, endosmose will here be a more general agent. Accordingly, the structure of these vessels is peculiarly adapted to this principle. They are very minute, full of valves, always of the same bore, run in straight lines, and pass through bodies which are called glands. In truth, their conditions are so similar to those of the lymphatic tubes in vegetables, that, without much use of figure, we may style this the vegetable department of the vascular system in animals. These glands are equivalents to the spongiolæ of the roots, or to the knots of the stems in grasses; and, being placed at convenient distances from each other, will exert an attractive and impulsive power, amply sufficient to circulate the contents of the vessels which pass through them. By afflux they will draw up, by impulse they will drive on, and when the weight of the rising column becomes too great for exosmose alone, endosmose comes in to make up for the deficiency.

Since then both endosmose and exosmose exist in the sound as well as diseased state of animals, it follows that inflammation is endosmose in a state of *disease*, or in a degree injurious to health. Now, one of the most ordinary causes of inflammation is the introduction of a foreign body into organic tissue, and one of the most essential conditions of endosmose is the presence of bodies of different densities in organic cavities; so that we are here furnished with a new reason why foreign *mild** bodies, denser than blood, produce inflammation or *hyper-endosmose*, and why hyper-endosmose will cease only on their removal. It has been already proved that the addition of a rarer fluid will weaken the endosmosmic power of the vesicular liquid, and it is well known that the introduction of water into an inflamed part is one of the most effectual means of affording it relief. Hence the use of baths, lotions, fomentations, and embrocations. The introduction of water has the double effect of diminishing the density of the fluids which produce hyper-endosmose, and, by occupying their place, of expelling from the diseased part the peccant humour. The *modus operandi* of endosmose in curing inflammation is, consequently, the same with that followed in effecting nutrition! However strange this assertion may appear it is an inference strictly drawn from the laws of endosmose. It must be recollected that organic solids are composed of vesicles filled with a denser fluid than

* *Mild* bodies are specified in contradistinction to such as may produce inflammation, by operating *chemically* upon the living fibre.

the blood which bathes their exterior surface;—that the blood thus situated must have a constant tendency to enter these vesicles, while their contents must also tend, but with less urgency, to pass out into the blood, or, in short, that each vesicle must exert a simultaneous endosmose and exosmose;—that the introduction of new matter into the vesicles constitutes the phenomena of *composition*, and the expulsion of the old matter *decomposition*, and that the conjunction of both phenomena constitutes the function of *nutrition*. It must again be recollected that the fluids contained within these vesicles may acquire either an unnatural density, or new chemical qualities, which shall increase their ordinary endosmose;—that, therefore, the blood, which is the exterior fluid, will tend to enter them in unusual quantity, and with unusual force;—that great turgidity will be the result of a stronger impulse than natural given to the venous blood; —that this increased power of introduction and expulsion, which is *morbid hyper-endosmose* or inflammation, tends to produce an entire renewal of the interior fluids of the vesicles; and that the period of this renewal is that of the complete cessation of morbid hyper-endosmose, if it have arisen from any alteration in the chemistry of the vesicular fluids, but if from their increased density, the hyper-endosmose will cease only when the proper consistence is restored by a sufficient introduction of a rarer substance. So that the *vis medicatrix naturæ*, of which we have heard so much and known so little, is nothing, whether in health or disease, but the continual play of a double power properly balanced, whereby fluids of different densities and constitutions are transferred from one organic cavity into another, and that the proper seat of inflammation is not in the walls of the vesicles or *solids*, but in the *fluids* which they circulate. It also appears that in a healthy state vital motion consists in the predominance of endosmose over exosmose, and that the degree of this predominance is fixed by the peculiar nature of the living being. When it becomes excessive we have diseases of excitement, and when it falls below *par* atonic maladies are produced. The predominance of exosmose is the entire destruction of the natural type of vital motion, and a state of local *hyper-exosmose* can only occur by the production of a new electrical condition, which would, by accelerating the motion of the heart, induce such a febrile state as a local hyper-endosmose would occasion. Thus does it become possible that two phenomena, of opposite characters, may be confounded under one general name—a name without a notion—*irritation*.

From the application of the same principle to the cure as well as the nature of disease, the author lays down an outline of the proper treatment to be followed in inflammation under the following heads, which we shall merely enunciate; 1st. The removal of the substance which causes hyper-endosmose; 2dly. General depletion, which, by lessening the general afflux, will diminish the local hyper-endosmose; 3dly. Local depletion, which gives relief in consequence of the blood removed, and by the derivation produced, or, in other words, by lowering the turgid state and forming an artificial afflux; 4thly. The establishment of a temporary endosmose superior to that of the disease, by blisters and rubefacients applied in the neighbourhood; 5thly. The application of water and all fluids rarer than the blood, by which the intensity of the endosmose may be lessened; 6thly. The introduction into the system by absorption, friction, &c. of such substances as will create an artificial general endosmose less dangerous than the disease; Lastly. The establishment of exosmose by surrounding the diseased parts with substances denser than the enclosed fluids, as poultices and acid lotions.

Absorption and exhalation were generally regarded as results of vascular action, until Magendie endeavoured to show that mere filtration will account for both. The experiments by which he arrived at this inference are ingenious, but all analogy is in opposition to his doctrine, and we doubt not but a few years will divest it of the artificial popularity, which the talents of its proposer have obtained for it. Dutrochet, as may be supposed, is equally inclined with ourselves to impugn this theory, and he accounts for the phenomena, which it attempts to explain, on his own principles. How, argues he, can an animal like the polypus, which has not a vascular system, continually absorb in virtue of capillary power; for when capillary cavities are once filled, no more fluid can be introduced. Not so in absorption produced by endosmose; it is constant, because it is accompanied by exhalation equally constant, which is the effect of exosmose. Every organ which is placed in a certain relation to fluids, will absorb and exhale; but these conditions are so necessary, that the absence of any one of them is fatal to the agency of all. In this manner can be explained a phenomenon, which has perplexed the most skilful physiologists,—we mean *elective attractions*. Thus, the chyle is the only fluid absorbed by the lacteals, although it and the fæces are intimately mixed and brush along their mouths in company; and why, because fæces produce exosmose, and milk, to which chyle bears a near similarity, produces endosmose?

In vegetables we have seen that secretion is conducted by endosmose; in animals it seems to be effected by the same power. The secreting organs of animals like those of vegetables are hollow bodies, through the walls of which the secretions are transmitted. In the molluscæ these hollow organs, or vesicles, are easily seen by the microscope, intermixed with blood-vessels and excretory ducts. The walls of these vesicles are pure *chemical filters*, which, under the influence of an electrical current, receive and modify certain elements of the common circulating fluid. The substance thus secreted is afterwards expelled by exosmose towards the excretory canals, or, if they be obstructed, towards the blood-vessels. So that when secretion is considered in this manner, it becomes a general principle to which nutrition itself seems to belong; for the nervous vesicle creates the nervous matter which fills it, the muscular vesicle the substance to which it owes its peculiarities, &c.; these vesicles then expel the substances they secrete, and these substances when expelled fall into the blood-vessels, which are the only excretories belonging to the *secretion of nutrition*.

The extent to which we have now drawn this article compels us to bring it to a close; but no one can be more sensible than we that the distinguished author, whose labours we have been reviewing, has been dismissed too soon, and that a protraction of the interview would be as instructive, as it would be interesting. But we trust that the sample we have exhibited will recommend the stock out of which it came, and that the inadequacy of our supply will induce the admirer to have recourse to the fund out of which we drew. For ourselves we can say, without being taxed with either adulation or venality, that Dutrochet is a physiologist of the first caliber, and his works performances of the first rank; that the simplicity of the agent which he has discovered can only be equalled by the extensive application it admits; and that the evidence, upon which its existence and laws are founded, must satisfy any impartial judgment. We doubt not that, like other discoverers, he has occasionally stretched his principles to their utmost length; but we can overlook such *fatherism* towards such a fondling, convinced, as we are, that the superior judgment of the parent will never suffer affection to *spoil* his child. Scarcely is there a point of difficulty in animal or vegetable physiology to which this principle does not reach, some which were perfectly unknown it perfectly explains, and on none, to which it can be applied, does it fail to shed considerable light. Circulation, absorption, nutrition, secretion, and even life itself seem indebted to its agency; and, should subsequent Inquirers find the results of their labours to correspond with those now laid before us, a new *Land of Promise* has been brought to light, which requires not a view from *Pisgah* to discover a rich variety of fruit.

